# C‐type lectin receptors of the Dectin‐1 cluster: Physiological roles and involvement in disease

**DOI:** 10.1002/eji.201847536

**Published:** 2019-11-13

**Authors:** Kazuya Tone, Mark H.T. Stappers, Janet A. Willment, Gordon D. Brown

**Affiliations:** ^1^ Aberdeen Fungal Group University of Aberdeen Institute of Medical Sciences Aberdeen Scotland; ^2^ Medical Research Council Centre for Medical Mycology University of Exeter Exeter Devon England

**Keywords:** Autoimmunity, C‐type lectin, Dectin‐1, Immunity

## Abstract

C‐type lectin receptors (CLRs) are essential for multicellular existence, having diverse functions ranging from embryonic development to immune function. One subgroup of CLRs is the Dectin‐1 cluster, comprising of seven receptors including MICL, CLEC‐2, CLEC‐12B, CLEC‐9A, MelLec, Dectin‐1, and LOX‐1. Reflecting the larger CLR family, the Dectin‐1 cluster of receptors has a broad range of ligands and functions, but importantly, is involved in numerous pathophysiological processes that regulate health and disease. Indeed, these receptors have been implicated in development, infection, regulation of inflammation, allergy, transplantation tolerance, cancer, cardiovascular disease, arthritis, and other autoimmune diseases. In this mini‐review, we discuss the latest advancements in elucidating the function(s) of each of the Dectin‐1 cluster CLRs, focussing on their physiological roles and involvement in disease.

## Graphical abstract

**The Dectin‐1 cluster of C‐type lectin receptors** (CLRs) are structurally related receptors that recognise a diverse array of ligands and are capable of intracellular signalling, inducing a wide variety of cellular responses. These CLRs are involved in numerous processes that are important for the regulation of health and disease

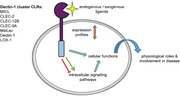

## Introduction

C‐type lectin receptors (CLRs) play essential roles in immunity and homeostasis 1. Comprising a family of more than 1000 proteins that each contain at least one C‐type lectin‐like domain, CLRs have been placed into 17 groups based on their structure and/or function. Originally named for their dependence on Ca^2+^ ions for carbohydrate recognition, many CLRs also possess conserved residues within their C‐type lectin‐like domains, such as the QPD (Gln‐Pro‐Asp) and EPN (Glu‐Pro‐Asn) motifs, which confer specificity for galactose and mannose‐containing carbohydrates, respectively. However, CLRs can lack these components and still recognise sugars, as well as broader repertoire of ligands such as proteins and lipids 1.

**Figure 1:**
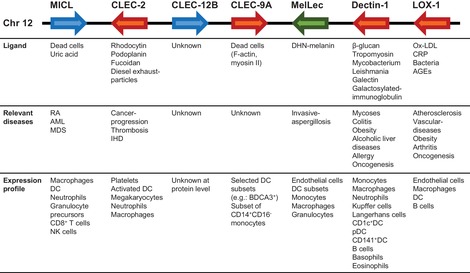
The Dectin‐1 cluster of C‐type lectin receptors. Shown is the genomic structure and transcriptional direction (arrows; blue = inhibitory CLR, red = activation CLR, green = unknown) of the Dectin‐1 cluster of C‐type lectin receptors found on human chromosome 12. The structure of the murine Dectin‐1 cluster on mouse chromosome 6 is similar (not shown). Also shown are selected ligands for each receptor, as well as the relevant diseases and/or pathological conditions that have been associated with these CLRs. The cellular expression profile for the human receptors is also shown. AGE, advanced glycation end‐products; AML, acute myeloid leukaemia; BDCA, Blood dendritic cell antigen; CD, cluster of differentiation; CRP, C‐reactive protein; DHN, 1,8‐dihydroxynaphthalene; IHD, ischemic heart diseases; MDS, myelodysplastic syndromes; Ox‐LDL, oxidised Low Density Lipoprotein; RA, rheumatoid arthritis.

The Dectin‐1 cluster forms part of one subgroup of CLRs (group V), which consists of seven structurally related receptors, all with a single carbohydrate recognition domain, that are all encoded in the same locus in both the mouse and human genome 2 (Fig. 1). The receptors in this cluster are of particular interest, as they were the first signalling CLRs to be identified on myeloid cells, and none appear to require calcium to recognise their ligands. These receptors, which include MICL, CLEC‐2, CLEC‐12B, CLEC‐9A, MelLec, Dectin‐1 and LOX‐1 (ordered based on genomic location), are involved in a broad range of physiological activities, from embryonic development to immunity. Importantly, the knowledge we are gaining from these receptors is opening exciting new possibilities for the diagnosis and treatment of disease. This mini‐review, an update on our previous review of the Dectin‐1 cluster 2, is aimed to provide an overview of each of these receptors, focusing on research published since 2016, highlighting the recent advancements made uncovering their functions.

### MICL

**Figure 2:**
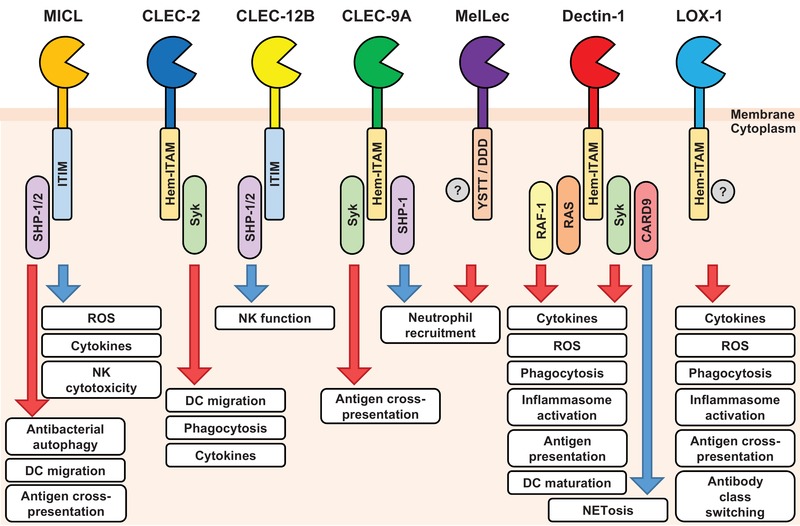
Representative signalling components utilized by the Dectin‐1 cluster of C‐type lectin receptors and selected cellular and immunological functions. The PAC‐Man shape indicates the carbohydrate recognition domain, and the cytoplasmic motifs present in each receptor are indicated. Red arrows indicate cellular functions that are stimulated following receptor ligation while blue arrows indicate cellular functions that are repressed following receptor ligation. Receptors have been ordered based on their genomic localisation. Detailed signalling pathways are not shown. CARD, caspase recruitment domain‐containing protein; NET, neutrophil extracellular traps; RAF, rapidly accelerated fibrosarcoma; Syk, spleen tyrosine kinase.

Myeloid inhibitory C‐type lectin‐like (MICL, CLEC‐12A, CLL‐1, DCAL‐2, KLRL‐1), as the name suggests, is primarily expressed by myeloid cells, including granulocytes and monocytes in humans and mice. MICL possesses an ITIM in its cytoplasmic tail, which can recruit Src homology region 2 domain‐containing phosphatase‐1 (SHP‐1) and SHP‐2, and through this pathway negatively regulates inflammatory cellular responses, including the respiratory burst 2 (Fig. 2). MICL has been implicated in antibacterial autophagy and in regulating antibacterial NK cell cytotoxicity as well as cytokine production, DC migration across the blood brain barrier, antigen cross‐presentation, and possibly a role in DC maturation 1, 2, 3.

Endogenous ligand(s) of MICL have been detected in various mouse tissues such as the heart, lung, liver, spleen, and kidney, and recent studies show that this receptor senses dead cells 1, 2. In fact, MICL recognises uric acid, a key danger signal for cell‐death‐induced immunity and MICL‐deficient mice exhibit hyper inflammatory responses to necrotic cells. MICL‐deficient mice also present with enhanced joint inflammation during a collagen antibody induced arthritis model, implicating the inhibitory functions of this receptor in the pathogenesis of rheumatoid arthritis. Although there are no genetic alterations linked to disease in humans, a subset of rheumatoid arthritis patients possess autoantibodies to MICL, which are able to exacerbate disease 4.

There has been considerable interest in human MICL in acute myeloid leukaemia (AML) and myelodysplastic syndromes (MDS), and this receptor represents a promising therapeutic and diagnostic target 5. MICL is highly expressed on leukaemia stem cells, but not on normal hematopoietic stem cells, and high levels of MICL expression is correlated to poor prognosis 5, 6. Immunotherapies for AML targeting MICL (including T cell recruiting bispecific antibodies and chimeric antigen receptor T cells) are being developed, and several are undergoing clinical trials 5.

### CLEC‐2

CLEC‐2 (CLEC‐1B) is expressed by platelets, megakaryocytes, and to a lesser extent by B cells and myeloid cells, including neutrophils, macrophages and subsets of DCs and has been largely studied in mouse models 1, 2. Several ligands for CLEC‐2 have been identified including rhodocytin (a protein present in snake venom), sulphated polysaccharides (such as fucoidan), diesel exhaust particles, and podoplanin (a mucin‐type glycoprotein), although the biochemical basis underlying some of these interactions is still unknown 1, 2. CLEC‐2 had been implicated in recognition of HIV, but this recognition was subsequently shown to be indirect, through viral incorporation of podoplanin. Like other receptors in the Dectin‐1 cluster, CLEC‐2 possesses a hemi‐ITAM in its cytoplasmic tail that mediates intracellular signalling though spleen tyrosine kinase (Syk)‐dependent pathways (Fig. 2).

CLEC‐2's expression on platelets has generated interest in its function in haemostasis and thrombosis. CLEC‐2 plays a minor role during normal haemostasis, facilitating platelet adhesion in the vasculature, but under inflammatory conditions, it has a more major role in preventing bleeding, although this may be organ specific 7. CLEC‐2 is implicated in thrombosis, particularly during inflammation, which causes upregulation of podoplanin on stromal cells and macrophages 7, 8. Interestingly, these activities of CLEC‐2 appear to be independent of its ability to induce intracellular signalling through the Syk pathway 9.

CLEC‐2 is involved in development, cancer, and immunity. In fact, CLEC‐2 plays a critical role in embryonic development, facilitating blood‐lymphatic separation, cerebrovascular patterning and integrity, lymph node and lung development, as well as platelet formation and the maintenance of hematopoietic stem cells in the bone marrow 1, 2, 10. In cancer, the interaction of CLEC‐2 with podoplanin‐expressing tumour cells promotes angiogenesis, tumour growth and metastasis, and may represent a target for therapy, if the side‐effects on the haemostatic functions of the receptor can be overcome 11. In contrast, expression of CLEC‐2 on some cancer cells, such as gastric cancer cells, may be protective 12.

CLEC‐2 is involved in DC migration and in the lymph node remodelling that is required for induction of adaptive immunity. During inflammation, CLEC‐2 can also regulate leukocyte recruitment and activation, including adaptive responses, through interactions with podoplanin expressed by these cells 13, 14, 15. Despite the considerable roles for CLEC‐2 that have been gleaned from mouse models, the direct involvement of this receptor in human disease is yet to be described. However, plasma levels of soluble human CLEC‐2, presumably generated by protease cleavage, as well as cellular expression levels are being explored as prognostic indicators of disease outcome (such as acute ischemic stroke 16 and cancer 17).

### CLEC‐12B

By comparison with the other receptors in the Dectin‐1 cluster, CLEC‐12B (Macrophage Antigen H, MAH) is poorly characterised. Expression of a full length and spliced variant transcript, lacking exon 4 (encoding part of the C‐type lectin‐like domain, and presumably nonfunctional), was detected by RT‐PCR in various human organs; however, only heart, kidney, liver, lung, spleen, and testis express the full length transcript 1, 2. At the protein level, CLEC‐12B has been detected on the human monocyte cell line, U937, following stimulation with phorbol 12‐myristate 13‐acetate, but the expression patterns of this receptor on mouse and human cells and tissues remain uncharacterised. CLEC‐12B contains a classical immunoreceptor tyrosine‐based inhibition motif in its cytoplasmic tail, that is able to recruit SHP‐1 and SHP‐2, and was able to inhibit NK cell function when transduced into these cells and following antibody crosslinking (Fig. 2).

Although its ligand(s) are unknown and its function is undefined, CLEC‐12B is thought to interact with caveolin‐1. In the context of disease, CLEC‐12B is upregulated along with other immunosuppressive genes during the transition from the acute to asymptomatic stage during HIV infections, and it is part of a set of negative regulators upregulated in Behçet's Syndrome 18. The presence of a polymorphism in *CLEC12B*, which may affect the secondary structure of the C‐type lectin‐like domain, has been linked in one family with predisposition to childhood cancer 19. Suggestively, both SHP‐1 and SHP‐2 have also been linked to cancer, including melanomas, but more work is required to define the physiological role of CLEC‐12B.

### CLEC‐9A

CLEC‐9A (DNGR‐1) is an endocytic receptor which recognises dead cells through exposed F‐actin, a process which is enhanced by myosin II 20. In humans, CLEC‐9A is expressed on immature BDCA3+ DCs and on a small subset on CD14+CD16‐ monocytes, and in the mouse, on CD8α+ conventional DC and plasmacytoid DCs. In fact, the expression profile of CLEC‐9A has promoted its use both as a cellular marker and lineage tracer.

Structurally, CLEC‐9A is very similar to Dectin‐1, containing a hem‐ITAM in its cytoplasmic tail that is able to induce intracellular signalling through Syk kinase (Fig. 2). Unlike Dectin‐1, however, CLEC‐9A does not appear to trigger cellular activation; rather induction of this signalling pathway promotes antigen cross‐presentation to CD8+ T cells through a poorly defined mechanism involving pH‐dependent conformational changes of the neck region of the receptor 21. The cross‐presentation activities of CLEC‐9A have been shown in mice to be required for promoting protective CD8+ T cell responses to some (e.g. vaccinia virus, HSV), but not all (e.g. RSV), viral infections 1. Notably, the ability of CLEC‐9A to promote cross‐presentation has prompted considerable interest in antigen targeting to this receptor to increase efficacy of cancer immunotherapies and vaccines 22.

Very recently, CLEC‐9A has been discovered to restrict neutrophil recruitment during inflammation to limit tissue damage 23. In mouse models of caerulein‐induced necrotizing pancreatitis and systemic *Candida albicans* infection 23, activation of CLEC‐9A by the cell death that occurred during inflammatory responses inhibited the production of the neutrophil‐recruiting chemokine, MIP‐2. Unexpectedly, this activity was found to be mediated through the recruitment of the inhibitory phosphatase, SHP‐1, to the cytoplasmic tail of CLEC‐9A (Fig. 2). The ability of CLEC‐9A to limit neutrophil recruitment has also been observed in a mouse model of *Mycobacterium tuberculosis* infection 24. In addition, there is also evidence from mice for involvement of this receptor in the regulation of inflammation during atherosclerosis 25. As yet, there is no link between CLEC‐9A and any human disease.

### MelLec

Melanin sensing C‐type Lectin receptor (MelLec, CLEC‐1, CLEC‐1A) was identified nearly two decades ago and is broadly expressed by endothelial cells in humans, mice, and rats. In humans and rats, this receptor is also expressed on myeloid cells, including various DC populations, monocytes, macrophages, and granulocytes 26. MelLec was recently shown to recognise 1, 8‐dihydroxynaphthalene melanin, an immunologically active component found in the cell wall of melanised fungi, such as *Aspergillus fumigatus* 27. In mice, loss of MelLec led to decreased survival and increased fungal burdens in a systemic model of *A. fumigatus* infection through a delay in neutrophil recruitment 27. Consistent with this observation, a polymorphism in the cytoplasmic tail of MelLec was associated with increased susceptibility to disseminated *Aspergillus* infections in stem‐cell transplant patients 27.

MelLec has also been implicated in modulating T cell function 26. In rats, the absence of MelLec led to exacerbated Th17 responses, which correlated with enhanced IL‐12p40 expression by DCs 26. Notably, decreased MelLec expression in human lung transplants was associated with increased levels of IL‐17A and chronic rejection 26. Similar findings were also observed in rat allograft models. This suggests that MelLec may play a role in the tolerogenic response to allografts, through recognition of an unknown endogenous ligand 26. How MelLec mediates its physiological functions is still unknown, although the receptor contains a YSST and tri‐acidic DDD motif in its cytoplasmic tail that could potentially mediate intracellular signalling 2 (Fig. 2).

### Dectin‐1

Dectin‐1 (CLEC‐7A) is one of the best characterised CLRs in mice and in humans and is predominantly expressed on myeloid cells, including monocytes, macrophages, dendritic cells, and neutrophils 28. Dectin‐1 is also expressed by B cells in humans and by some subsets of T cells 1. There is also some evidence for expression on other cell types, including epithelial cells 1, 29. There are two major isoforms of Dectin‐1 (one of which lacks the stalk region), and these show cell (and mouse strain) specific patterns of expression. Dectin‐1 recognises β‐glucans, carbohydrates commonly found in the cell walls of plants and fungi, but has also been reported to recognise tropomyosin (found in arthropods) and unidentified ligand(s) in mycobacteria and Leishmania 28, 30, 31. Several endogenous ligands have also been identified including vimentin, galactosylated immunoglobulins, and galectins 28, 32.

Ligand recognition by Dectin‐1 triggers intracellular signalling through a hem‐ITAM in the cytoplasmic tail of the receptor that induces multiple downstream pathways, including Raf‐1 and Syk/CARD9 (Fig. 2). Signalling from Dectin‐1 can induce or regulate numerous cellular responses, including phagocytosis, the respiratory burst, neutrophil extracellular trap formation, autophagy, DC maturation and antigen presentation, inflammasome activation (including the NLRP3 and the non‐canonical caspase‐8 inflammasomes), and the production of eicosanoids, cytokines, and chemokines 28. Dectin‐1 is also capable of modulating the cellular responses induced by other pathogen pattern recognition receptors, can directly induce innate immune memory, and influence the development of CD4 and CD8 T cells and B cell responses 1, 33, 34.

Dectin‐1 has been most studied in the context of anti‐fungal immunity using mouse models. Indeed, through its ability to recognise β‐1,3‐linked glucan, Dectin‐1 is required to drive protective host responses to many pathogenic fungal species, including *Aspergillus, Candida, Pneumocystis*, although its involvement may depend on particular strains of these organisms. Importantly, in humans, polymorphisms of Dectin‐1 are associated with increased susceptibility to fungal disease 28. The functions of Dectin‐1 are also important for maintaining gastrointestinal homeostasis and can exacerbate the severity of colitis, through recognition specific fungi in the microbiota as well as food derived β‐glucans 35, 36, 37. Interestingly, Dectin‐1 responses have been implicated in the pathogenesis of obesity 38 and alcoholic liver disease, following intestinal release of fungal β‐glucans into the bloodstream 39.

More recently, Dectin‐1 has been implicated in allergy and cancer. Although Dectin‐1 is known to promote Th1 and Th17 immunity, this CLR can also drive Th2 responses, in part through the production of prostaglandin E2 33. Indeed, through this and other pathways, including regulation of IL‐22 and IL‐33, Dectin‐1 has been linked to the pathology of allergic responses in both mouse models and in humans 30, 40. Dectin‐1 has dual functions in cancer, either promoting or preventing oncogenesis. For example, in pancreatic ductal adenocarcinoma, Dectin‐1‐mediated recognition of tumour‐associated Galectin‐9 supresses protective T cell responses 32. In contrast, Dectin‐1‐mediated recognition of N‐glycan structures on tumour cells can lead to activation of the tumoricidal activities of NK‐cells. Dectin‐1 can also mediate protective activities by supressing TLR4‐mediated inflammation and by promoting Th9 immunity 1, 34.

### LOX‐1

Lectin‐like oxidised low density lipoproteins (LDL) receptor‐1 (LOX‐1, OLR‐1, CLEC‐8A) is expressed on endothelial cells but also by a variety of other cell types including leukocyte populations (such as macrophages, monocytes, DCs, and B cells), smooth muscle cells, neurons, and fibroblasts 41. Although normally expressed at low levels, in part through regulation by casein kinase 2‐interacting protein‐1 42, LOX‐1 is rapidly upregulated by a variety of factors, particularly pro‐atherogenic stimuli including inflammatory cytokines and modified LDL. LOX‐1 can be cleaved by ADAM10 proteases to produce a soluble form (sLOX‐1) than is detectable in the serum, and N‐terminal fragments that remain bound in the membrane 43. LOX‐1 is able to recognise a broad range of ligands including bacterial products, C‐reactive protein, and advanced glycation end products, but is best known for its ability to bind oxidised LDL (ox‐LDL). LOX‐1 can internalize its ligands through a cytoplasmic tripeptide (DDL) motif, and induce intracellular signalling that results in a variety of cellular responses including the production of ROS, chemokines and cytokines, upregulation of adhesion molecules, apoptosis, as well as activation of the NALP3 inflammasome and NF‐*κ*B 44. How LOX‐1 mediates intracellular signalling is unclear, but recent evidence implicates the membrane N‐terminal fragments in these activities, and their regulation by the signal peptide peptidase–like 2a and b (SPPL2a/b) 43.

LOX‐1 has been extensively studied in the context of atherosclerosis and associated vascular diseases, including hypertension and stroke. Indeed, data from mouse models has convincingly demonstrated the role of LOX‐1 in numerous pro‐atherogenic activities including macrophage foam cell and plaque formation, endothelial dysfunction (such as increased ROS leading to activation of NF‐κB and induction of adhesion molecules and apoptosis), proliferation of vascular smooth muscle cells, platelet aggregation, and leukocyte recruitment 41. Notably, in humans, polymorphisms and alternative splice variants of LOX‐1 gene are associated with either promotion or protection from disease 45. Moreover, the levels of sLOX‐1 can be used as a prognostic and diagnostic marker for cardiovascular disease, and possibly stoke, in patients 41, 44, 46. Given its detrimental activities, there is considerable interest in developing novel therapeutics that target LOX‐1. Several drugs already in clinical use for treating cardiovascular disease appear to affect LOX‐1, including statins, which disrupts LOX1‐mediated recognition of oxLDL 41.

In the immune system, LOX‐1 is able to regulate inflammatory responses to bacterial (including sepsis) and fungal pathogens. In fact, there is emerging evidence from mouse models that LOX‐1 may represent a therapeutic target to treat *Aspergillus*‐mediated keratitis 47. LOX‐1 is also involved in antigen cross‐presentation (in part through the recognition of dead cells) and can promote B cell differentiation and antibody class switching. Moreover, targeting LOX‐1 can promote antigen‐specific T cell responses 48.

LOX‐1 is involved in the pathophysiology of arthritis and cancer. In mouse models of arthritis and osteoarthritis, deletion of LOX‐1 (which is upregulated on chondrocytes and other cells in affected joints) was shown to protect against disease 49, 50. In humans, sLOX‐1 has been proposed as a biomarker for diagnosis and evaluation of disease activity. Changes in lipid metabolism are associated with oncogenesis, and the activities of LOX‐1 (which is upregulated in a wide range of cancers) in this context have been linked to promoting the development of cancer, including angiogenesis, tumour invasion, and metastasis 51. Expression levels of LOX‐1 have been proposed to have prognostic and therapeutic potential 52, 53.

## Conclusion

Receptors of the Dectin‐1 cluster have a wide range of functions and are involved in numerous diseases. In the last two decades, many important discoveries have been made regarding these receptors that have therapeutic implications. Indeed, characterisation of their structures, the identification of their ligands, and their signal transduction pathways has already led to new prognostic and diagnostic markers and novel immunotherapeutic approaches. It will be exciting to see how our ever‐expanding knowledge of this C‐type lectin subfamily is used to tackle disease in the future.

## Conflict of interest

The authors declare no commercial or financial conflict of interest.

**Abbreviations**
AML: acute myeloid leukaemia
CLR: C‐type lectin receptor
LDL: low density lipoproteins
MDS: myelodysplastic syndromes
MICL: myeloid inhibitory C‐type lectin‐like

## References

[1] Brown, G. D. , Willment, J. A. and Whitehead, L. , C‐type lectins in immunity and homeostasis. Nat. Rev. Immunol. 2018 18: 374–389. 29581532 10.1038/s41577-018-0004-8

[2] Plato, A. , Willment, J. A. and Brown, G. D. , C‐type lectin‐like receptors of the dectin‐1 cluster: ligands and signaling pathways. Int. Rev. Immunol. 2013 32: 134–156. 23570314 10.3109/08830185.2013.777065 PMC3634610

[3] Sagar, D. , Singh, N. P. , Ginwala, R. , Huang, X. , Philip, R. , Nagarkatti, M. , Nagarkatti, P. et al., Antibody blockade of CLEC12A delays EAE onset and attenuates disease severity by impairing myeloid cell CNS infiltration and restoring positive immunity. Sci. Rep. 2017 7: 2707. 28578388 10.1038/s41598-017-03027-x PMC5457463

[4] Redelinghuys, P. , Whitehead, L. , Augello, A. , Drummond, R. A. , Levesque, J. M. , Vautier, S. , Reid, D. M. et al., MICL controls inflammation in rheumatoid arthritis. Ann. Rheum. Dis. 2016 75: 1386–1391. 26275430 10.1136/annrheumdis-2014-206644 PMC4941174

[5] Morsink, L. M. , Walter, R. B. and Ossenkoppele, G. J. , Prognostic and therapeutic role of CLEC12A in acute myeloid leukemia. Blood Rev. 2019 34: 26–33. 30401586 10.1016/j.blre.2018.10.003

[6] Toft‐Petersen, M. , Nederby, L. , Kjeldsen, E. , Kerndrup, G. B. , Brown, G. D. , Hokland, P. and Stidsholt Roug, A. , Unravelling the relevance of CLEC12A as a cancer stem cell marker in myelodysplastic syndrome. Br. J. Haematol. 2016 175: 393–401. 27612176 10.1111/bjh.14270 PMC5091626

[7] Rayes, J. , Watson, S. P. and Nieswandt, B. , Functional significance of the platelet immune receptors GPVI and CLEC‐2. J. Clin. Invest. 2019 129: 12–23. 30601137 10.1172/JCI122955 PMC6307936

[8] Payne, H. , Ponomaryov, T. , Watson, S. P. and Brill, A. , Mice with a deficiency in CLEC‐2 are protected against deep vein thrombosis. Blood 2017 129: 2013–2020. 28104688 10.1182/blood-2016-09-742999 PMC5408561

[9] Haining, E. J. , Cherpokova, D. , Wolf, K. , Becker, I. C. , Beck, S. , Eble, J. A. , Stegner, D. et al., CLEC‐2 contributes to hemostasis independently of classical hemITAM signaling in mice. Blood 2017 130: 2224–2228. 28835437 10.1182/blood-2017-03-771907

[10] Tsukiji, N. , Inoue, O. , Morimoto, M. , Tatsumi, N. , Nagatomo, H. , Ueta, K. , Shirai, T. et al., Platelets play an essential role in murine lung development through Clec‐2/podoplanin interaction. Blood 2018 132: 1167–1179. 29853539 10.1182/blood-2017-12-823369

[11] Tsukiji, N. , Osada, M. , Sasaki, T. , Shirai, T. , Satoh, K. , Inoue, O. , Umetani, N. et al., Cobalt hematoporphyrin inhibits CLEC‐2‐podoplanin interaction, tumor metastasis, and arterial/venous thrombosis in mice. Blood Adv. 2018 2: 2214–2225. 30190281 10.1182/bloodadvances.2018016261 PMC6134222

[12] Wang, L. , Yin, J. , Wang, X. , Shao, M. , Duan, F. , Wu, W. , Peng, P. et al., C‐type lectin‐like receptor 2 suppresses AKT signaling and invasive activities of gastric cancer cells by blocking expression of phosphoinositide 3‐kinase subunits. Gastroenterology 2016 150: 1183–1195.e1116. 26855187 10.1053/j.gastro.2016.01.034

[13] Lax, S. , Rayes, J. , Wichaiyo, S. , Haining, E. J. , Lowe, K. , Grygielska, B. , Laloo, R. et al., Platelet CLEC‐2 protects against lung injury via effects of its ligand podoplanin on inflammatory alveolar macrophages in the mouse. Am. J. Physiol. Lung Cell. Mol. Physiol. 2017 313: L1016–l1029. 28839100 10.1152/ajplung.00023.2017 PMC5814702

[14] Nylander, A. N. , Ponath, G. D. , Axisa, P. P. , Mubarak, M. , Tomayko, M. , Kuchroo, V. K. , Pitt, D. et al., Podoplanin is a negative regulator of Th17 inflammation. JCI Insight 2017 2: e92321. 10.1172/jci.insight.92321 PMC5621890 28878118

[15] Rayes, J. , Lax, S. , Wichaiyo, S. , Watson, S. K. , Di, Y. , Lombard, S. , Grygielska, B. et al., The podoplanin‐CLEC‐2 axis inhibits inflammation in sepsis. Nat. Commun. 2017 8: 2239. 29269852 10.1038/s41467-017-02402-6 PMC5740111

[16] Wu, X. , Zhang, W. , Li, H. , You, S. , Shi, J. , Zhang, C. , Shi, R. et al., Plasma C‐type lectin‐like receptor 2 as a predictor of death and vascular events in patients with acute ischemic stroke. Eur. J. Neurol. 2019: 26: 1334–1340. 10.1111/ene.13984 31081579

[17] Xiong, Y. , Liu, L. , Xia, Y. , Wang, J. , Xi, W. , Bai, Q. , Qu, Y. et al., High CLEC‐2 expression associates with unfavorable postoperative prognosis of patients with clear cell renal cell carcinoma. Oncotarget 2016 7: 63661–63668. 27564117 10.18632/oncotarget.11606 PMC5325393

[18] Oguz, A. K. , Yilmaz, S. T. , Oygur, C. S. , Candar, T. , Sayin, I. , Kilicoglu, S. S. , Ergun, I. et al., Behcet's: a disease or a syndrome? Answer from an expression profiling study. PLoS One 2016 11: e0149052. 26890122 10.1371/journal.pone.0149052 PMC4758705

[19] Derpoorter, C. , Vandepoele, K. , Diez‐Fraile, A. , Vandemeulebroecke, K. , De Wilde, B. , Speleman, F. , Van Roy, N. et al., Pinpointing a potential role for CLEC12B in cancer predisposition through familial exome sequencing. Pediatr. Blood Cancer 2019 66: e27513. 30350915 10.1002/pbc.27513

[20] Schulz, O. , Hanc, P. , Bottcher, J. P. , Hoogeboom, R. , Diebold, S. S. , Tolar, P. and Reis, E. S. C. , Myosin II synergizes with F‐actin to promote DNGR‐1‐dependent cross‐presentation of dead cell‐associated antigens. Cell Rep. 2018 24: 419–428. 29996102 10.1016/j.celrep.2018.06.038 PMC6057488

[21] Hanc, P. , Schulz, O. , Fischbach, H. , Martin, S. R. , Kjaer, S. and Reis e Sousa, C. , A pH‐ and ionic strength‐dependent conformational change in the neck region regulates DNGR‐1 function in dendritic cells. EMBO J. 2016 35: 2484–2497. 27753620 10.15252/embj.201694695 PMC5109244

[22] Zeng, B. , Middelberg, A. P. , Gemiarto, A. , MacDonald, K. , Baxter, A. G. , Talekar, M. , Moi, D. et al., Self‐adjuvanting nanoemulsion targeting dendritic cell receptor Clec9A enables antigen‐specific immunotherapy. J. Clin. Invest. 2018 128: 1971–1984. 29485973 10.1172/JCI96791 PMC5919883

[23] Del Fresno, C. , Saz‐Leal, P. , Enamorado, M. , Wculek, S. K. , Martinez‐Cano, S. , Blanco‐Menendez, N. , Schulz, O. et al., DNGR‐1 in dendritic cells limits tissue damage by dampening neutrophil recruitment. Science 2018 362: 351–356. 30337411 10.1126/science.aan8423

[24] Cheng, A. C. , Yang, K. Y. , Chen, N. J. , Hsu, T. L. , Jou, R. , Hsieh, S. L. and Tseng, P. H. , CLEC9A modulates macrophage‐mediated neutrophil recruitment in response to heat‐killed Mycobacterium tuberculosis H37Ra. PLoS One 2017 12: e0186780. 29065139 10.1371/journal.pone.0186780 PMC5655532

[25] Haddad, Y. , Lahoute, C. , Clement, M. , Laurans, L. , Metghalchi, S. , Zeboudj, L. , Giraud, A. et al., The dendritic cell receptor DNGR‐1 promotes the development of atherosclerosis in mice. Circ. Res. 2017 121: 234–243. 28607102 10.1161/CIRCRESAHA.117.310960

[26] Lopez Robles, M. D. , Pallier, A. , Huchet, V. , Le Texier, L. , Remy, S. , Braudeau, C. , Delbos, L. et al., Cell‐surface C‐type lectin‐like receptor CLEC‐1 dampens dendritic cell activation and downstream Th17 responses. Blood Adv. 2017 1: 557–568. 29296975 10.1182/bloodadvances.2016002360 PMC5728597

[27] Stappers, M. H. T. , Clark, A. E. , Aimanianda, V. , Bidula, S. , Reid, D. M. , Asamaphan, P. , Hardison, S. E. et al., Recognition of DHN‐melanin by a C‐type lectin receptor is required for immunity to Aspergillus. Nature 2018 555: 382–386. 29489751 10.1038/nature25974 PMC5857201

[28] Brown, G. D. and Crocker, P. R. , Lectin receptors expressed on myeloid cells. Microbiol. Spectr. 2016 4: MCHD‐0036‐2016. 10.1128/microbiolspec.MCHD-0036-2016 27780012

[29] Sun, W. K. , Lu, X. , Li, X. , Sun, Q. Y. , Su, X. , Song, Y. , Sun, H. M. et al., Dectin‐1 is inducible and plays a crucial role in Aspergillus‐induced innate immune responses in human bronchial epithelial cells. Eur. J. Clin. Microbiol. Infect. Dis. 2012 31: 2755–2764. 22562430 10.1007/s10096-012-1624-8

[30] Gour, N. , Lajoie, S. , Smole, U. , White, M. , Hu, D. , Goddard, P. , Huntsman, S. et al., Dysregulated invertebrate tropomyosin‐dectin‐1 interaction confers susceptibility to allergic diseases. Sci. Immunol. 2018 3: eaam9841. 29475849 10.1126/sciimmunol.aam9841 PMC5956913

[31] Lima‐Junior, D. S. , Mineo, T. W. P. , Calich, V. L. G. and Zamboni, D. S. , Dectin‐1 activation during leishmania amazonensis phagocytosis prompts syk‐dependent reactive oxygen species production to trigger inflammasome assembly and restriction of parasite replication. J. Immunol. 2017 199: 2055–2068. 28784846 10.4049/jimmunol.1700258

[32] Daley, D. , Mani, V. R. , Mohan, N. , Akkad, N. , Ochi, A. , Heindel, D. W. , Lee, K. B. et al., Dectin 1 activation on macrophages by galectin 9 promotes pancreatic carcinoma and peritumoral immune tolerance. Nat. Med. 2017 23: 556–567. 28394331 10.1038/nm.4314 PMC5419876

[33] Kaisar, M. M. M. , Ritter, M. , Del Fresno, C. , Jonasdottir, H. S. , van der Ham, A. J. , Pelgrom, L. R. , Schramm, G. et al., Dectin‐1/2‐induced autocrine PGE2 signaling licenses dendritic cells to prime Th2 responses. PLoS Biol. 2018 16: e2005504. 29668708 10.1371/journal.pbio.2005504 PMC5927467

[34] Zhao, Y. , Chu, X. , Chen, J. , Wang, Y. , Gao, S. , Jiang, Y. , Zhu, X. et al., Dectin‐1‐activated dendritic cells trigger potent antitumour immunity through the induction of Th9 cells. Nat. Commun. 2016 7: 12368. 27492902 10.1038/ncomms12368 PMC4980454

[35] Drummond, R. A. , Dambuza, I. M. , Vautier, S. , Taylor, J. A. , Reid, D. M. , Bain, C. C. , Underhill, D. M. et al., CD4(+) T‐cell survival in the GI tract requires dectin‐1 during fungal infection. Mucosal. Immunol. 2016 9: 492–502. 26349660 10.1038/mi.2015.79 PMC4677461

[36] Kamiya, T. , Tang, C. , Kadoki, M. , Oshima, K. , Hattori, M. , Saijo, S. , Adachi, Y. et al., beta‐Glucans in food modify colonic microflora by inducing antimicrobial protein, calprotectin, in a Dectin‐1‐induced‐IL‐17F‐dependent manner. Mucosal. Immunol. 2018 11: 763–773. 29068000 10.1038/mi.2017.86

[37] Takagawa, T. , Kitani, A. , Fuss, I. , Levine, B. , Brant, S. R. , Peter, I. , Tajima, M. et al., An increase in LRRK2 suppresses autophagy and enhances Dectin‐1‐induced immunity in a mouse model of colitis. Sci. Transl. Med. 2018 10: eaan8162. 29875204 10.1126/scitranslmed.aan8162 PMC6636639

[38] Castoldi, A. , Andrade‐Oliveira, V. , Aguiar, C. F. , Amano, M. T. , Lee, J. , Miyagi, M. T. , Latancia, M. T. et al., Dectin‐1 activation exacerbates obesity and insulin resistance in the absence of MyD88. Cell Rep. 2017 19: 2272–2288. 28614714 10.1016/j.celrep.2017.05.059 PMC9261359

[39] Yang, A. M. , Inamine, T. , Hochrath, K. , Chen, P. , Wang, L. , Llorente, C. , Bluemel, S. et al., Intestinal fungi contribute to development of alcoholic liver disease. J. Clin. Invest. 2017 127: 2829–2841. 28530644 10.1172/JCI90562 PMC5490775

[40] Ito, T. , Hirose, K. , Norimoto, A. , Tamachi, T. , Yokota, M. , Saku, A. , Takatori, H. et al., Dectin‐1 plays an important role in house dust mite‐induced allergic airway inflammation through the activation of CD11b+ dendritic cells. J. Immunol. 2017 198: 61–70. 27852745 10.4049/jimmunol.1502393

[41] Jin, P. and Cong, S. , LOX‐1 and atherosclerotic‐related diseases. Clin. Chim. Acta 2019 491: 24–29. 30639239 10.1016/j.cca.2019.01.006

[42] Fan, J. , Liu, L. , Liu, Q. , Cui, Y. , Yao, B. , Zhang, M. , Gao, Y. et al., CKIP‐1 limits foam cell formation and inhibits atherosclerosis by promoting degradation of Oct‐1 by REGgamma. Nat. Commun. 2019 10: 425. 30683852 10.1038/s41467-018-07895-3 PMC6347643

[43] Mentrup, T. , Theodorou, K. , Cabrera‐Cabrera, F. , Helbig, A. O. , Happ, K. , Gijbels, M. , Gradtke, A. C. et al., Atherogenic LOX‐1 signaling is controlled by SPPL2‐mediated intramembrane proteolysis. J. Exp. Med. 2019 216: 807–830. 30819724 10.1084/jem.20171438 PMC6446863

[44] Tian, K. , Ogura, S. , Little, P. J. , Xu, S. W. and Sawamura, T. , Targeting LOX‐1 in atherosclerosis and vasculopathy: current knowledge and future perspectives. Ann. N. Y. Acad. Sci. 2019 1443: 34–53. 30381837 10.1111/nyas.13984

[45] Rizzacasa, B. , Morini, E. , Pucci, S. , Murdocca, M. , Novelli, G. and Amati, F. , LOX‐1 and its splice variants: a new challenge for atherosclerosis and cancer‐targeted therapies. Int. J. Mol. Sci. 2017 18: 290. 10.3390/ijms18020290 PMC5343826 28146073

[46] Li, X. M. , Jin, P. P. , Xue, J. , Chen, J. , Chen, Q. F. , Luan, X. Q. , Zhang, Z. R. et al., Role of sLOX‐1 in intracranial artery stenosis and in predicting long‐term prognosis of acute ischemic stroke. Brain Behav. 2018 8: e00879. 29568681 10.1002/brb3.879 PMC5853620

[47] Sun, Q. , Li, C. , Lin, J. , Peng, X. , Wang, Q. , Jiang, N. , Xu, Q. et al., Celastrol ameliorates Aspergillus fumigatus keratitis via inhibiting LOX‐1. Int. Immunopharmacol. 2019 70: 101–109. 30798158 10.1016/j.intimp.2019.02.017

[48] Zurawski, G. , Zurawski, S. , Flamar, A. L. , Richert, L. , Wagner, R. , Tomaras, G. D. , Montefiori, D. C. et al., Targeting HIV‐1 Env gp140 to LOX‐1 elicits immune responses in rhesus macaques. PLoS One 2016 11: e0153484. 27077384 10.1371/journal.pone.0153484 PMC4831750

[49] Hashimoto, K. , Mori, S. , Oda, Y. , Nakano, A. , Sawamura, T. and Akagi, M. , Lectin‐like oxidized low density lipoprotein receptor 1‐deficient mice show resistance to instability‐induced osteoarthritis. Scand. J. Rheumatol. 2016 45: 412–422. 26901593 10.3109/03009742.2015.1135979

[50] Hashimoto, K. , Oda, Y. , Nakagawa, K. , Ikeda, T. , Ohtani, K. and Akagi, M. , LOX‐1 deficient mice show resistance to zymosan‐induced arthritis. Eur. J. Histochem. 2018 62: 2847. 29569871 10.4081/ejh.2018.2847 PMC5806501

[51] Balzan, S. and Lubrano, V. , LOX‐1 receptor: a potential link in atherosclerosis and cancer. Life Sci. 2018 198: 79–86. 29462603 10.1016/j.lfs.2018.02.024

[52] Condamine, T. , Dominguez, G. A. , Youn, J. I. , Kossenkov, A. V. , Mony, S. , Alicea‐Torres, K. , Tcyganov, E. et al., Lectin‐type oxidized LDL receptor‐1 distinguishes population of human polymorphonuclear myeloid‐derived suppressor cells in cancer patients. Sci. Immunol. 2016 1: AAF8943. 28417112 10.1126/sciimmunol.aaf8943 PMC5391495

[53] Murdocca, M. , Mango, R. , Pucci, S. , Biocca, S. , Testa, B. , Capuano, R. , Paolesse, R. et al., The lectin‐like oxidized LDL receptor‐1: a new potential molecular target in colorectal cancer. Oncotarget 2016 7: 14765–14780. 26895376 10.18632/oncotarget.7430 PMC4924750

